# Co-infection of Four Novel Mycoviruses from Three Lineages Confers Hypovirulence on Phytopathogenic Fungus *Ustilaginoidea virens*

**DOI:** 10.1186/s12284-024-00721-z

**Published:** 2024-07-16

**Authors:** Yu Fan, Wenhua Zhao, Xiaolin Tang, Mei Yang, Yingqing Yang, Zixuan Zhang, Baoping Cheng, Erxun Zhou, Zhenrui He

**Affiliations:** 1 Guangdong Province Key Laboratory of Microbial Signals and Disease Control, College of Plant Protection, South China Agricultural University, Guangzhou, 510642 China; 2 Institute of Plant Protection, Jiangxi Academy of Agricultural Sciences, Nanchang, 330200 China; 3 Institute of Plant Protection, Guangdong Academy of Agricultural Sciences/Guangdong Provincial Key Laboratory of High Technology for Plant Protection/Key Laboratory of Green Prevention and Control On Fruits and Vegetables in South China, Ministry of Agriculture and Rural Affairs, Guangdong, 510642 China

**Keywords:** Mycovirus, Rice false smut, Co-infection, Biocontrol

## Abstract

Rice false smut caused by *Ustilaginoidea virens* has become one of the most important diseases of rice. Mycoviruses are viruses that can infect fungi with the potential to control fungal diseases. However, little is known about the biocontrol role of hypoviruses in *U. virens*. In this study, we revealed that the hypovirulence-associated *U. virens* strain Uv325 was co-infected by four novel mycoviruses from three lineages, designated Ustilaginoidea virens RNA virus 16 (UvRV16), Ustilaginoidea virens botourmiavirus virus 8 (UvBV8), Ustilaginoidea virens botourmiavirus virus 9 (UvBV9), and Ustilaginoidea virens narnavirus virus 13 (UvNV13), respectively. The *U. virens* strain co-infected by four mycoviruses showed slower growth rates, reduced conidial yield, and attenuated pigmentation. We demonstrated that UvRV16 was not only the major factor responsible for the hypovirulent phenotype in *U. vriens*, but also able to prevent *U. virens* to accumulate more mycotoxin, thereby weakening the inhibitory effects on rice seed germination and seedling growth. Additionally, we indicated that UvRV16 can disrupt the antiviral response of *U. virens* by suppressing the transcriptional expression of multiple genes involved in autophagy and RNA silencing. In conclusion, our study provided new insights into the biological control of rice false smut.

**Supplementary Information:**

The online version contains supplementary material available at 10.1186/s12284-024-00721-z.

## Introduction

Mycoviruses are a class of viruses that infect and replicate in fungi or oomycetes and are widespread in all major taxa of fungi (Ghabrial and Suzuki 2009; Xie and Jiang 2014; Ghabrial et al. 2015). Since the first definitive report of the cultivated button mushroom *Agaricus bisporus* infected by mycoviruses in 1962 (Hollings 1962), the exploration of mycoviruses has been ongoing for 61 years. According to the latest list of mycoviruses proposed by the International Committee on Taxonomy of Viruses (ICTV), mycoviruses usually have double-stranded (dsRNA) or single-stranded (ssRNA) genomes (Kotta-Loizou and Coutts 2017), however, DNA viruses have also been identified in recent years in *Sclerotinia sclerotiorum* and *Fusarium graminearum* (Yu et al. 2010; Li et al. 2020). Unlike most plant, animal and bacterial viruses, the majority of mycoviruses do not usually attack fungal cells and are asymptomatic in vitro (Xie and Jiang 2014; Ghabrial et al. 2015). Notably, some mycoviruses can reduce the pathogenicity of their fungal hosts, known as hypoviruses, which are an important biocontrol resource because of their hypovirulent characteristics. The most successful instance is the application of Cryphonectria hypovirus 1 (CHV1) in Europe to control the chestnut blight caused by *Cryphonectria parasitica* (Anagnostakis 1982). In recent years, Zhang et al. have discovered that rapeseed stem rot caused by *Sclerotinia sclerotiorum* can be effectively controlled by spraying Sclerotinia sclerotiorum hypovirulence-associated DNA virus 1 (SsHADV-1)-infected strain DT-8 in the field (Zhang et al. 2020). SsHADV-1 can convert its fungal host *S. sclerotiorum* from a typical necrotrophic pathogen to a beneficial endophytic fungus. Furthermore, there are some reports showing that mycoviruses can attenuate fungal pathogenicity, reduce sporulation, alter colony morphology and cause poor mycelial growth (Liu et al. 2022). Therefore, exploring the diversity and biological characteristics of mycoviruses can provide new resources and new insights into the biological control of plant fungal diseases caused by phytopathogenic fungi.

Rice false smut (RFS), caused by ascomycetous fungus *Ustilaginoidea virens* (Cooke) Takahashi (teleomorph: *Villosiclava virens*), is a devastating disease in rice-growing regions worldwide. With the widespread cultivation of high-yielding varieties and hybrids, excessive use of chemical fertilizers, and marked changes in global and regional climates, RFS has become one of the most devastating cereal diseases in rice-growing areas worldwide (Sun et al. 2020; He et al. 2022; Zhang et al. 2014b). *U. virens* not only poses serious threats to global food security, but its mycotoxin also poses threats the health of humans and animals (Koiso et al. 1994; Wang et al. 2017). Mycotoxins are important in the pathogenicity of phytopathogenic fungi and usually play an important role in pathogen colonization and biological processes (Hu et al. 2020). In *U. virens*, mycotoxins can inhibit the growth and development of rice spikes and the accumulation of total sugars, as well as act as pathogenic factors to help *U. virens* infect rice (Li et al. 2019; Meng et al. 2019; Sun et al. 2020; Fu et al. 2022). *U. virens* contaminates rice grain by generating two major types of mycotoxins during infection, including the cyclopeptide ustiloxins and the polyketide ustilaginoidins (Guo et al. 2012; Lu et al. 2014, 2015). In *Fusarium graminearum*, the production of deoxynivalenol (DON) acts on host plant cell walls, chloroplasts and endoplasmic reticulum and plays a key role in wheat spike, pathogenesis and colonization (Wang et al. 2020). The current main measure to control RFS is still environmentally unfriendly fungicide, which not only results in increased antifungal resistance, but also threatens environmental safety and human health. Therefore, the mycoviruses that infect *U. virens* have attracted our attention for their high efficiency and persistency. To date, only the genome characterization of dsRNA viruses has been reported in *U. virens*, including Ustilaginoidea virens RNA virus 1-6 (UvRV1-6) (Zhang et al. 2013a; Zhong et al. 2014a, b; Jiang et al. 2015; Zhong et al. 2017), Ustilaginoidea virens partitivirus 1-4 (UvPV1-4) (Zhang et al. 2013b; Zhong et al. 2014c, 2014a; Jiang et al. 2014), Ustilaginoidea virens nonsegmented virus 1-2 (UvNV1-2) (Zhang et al. 2014a, 2018) and Ustilaginoidea virens unassigned RNA virus HNND-1 (UvURV-HNND-1) (Zhu et al. 2015). Furthermore, our laboratory previously identified 593 mycovirus-related contigs in 182 *U. virens* strains by metatranscriptomic sequencing (He et al. 2022). However, there are no reports of hypoviruses in *U. virens*, which attracts us to explore efficient and environmentally friendly novel hypoviruses to control RFS.

Viral co-infections are a common phenomenon in animals and plants and are currently being found in fungi. The interactions between co-infecting mycoviruses may cause different degrees of viral disease symptoms in host fungi via synergistic or antagonistic mechanisms (Hillman et al. 2018; Thapa and Roossinck 2019). For instance, the hypovirulent *Sclerotinia sclerotiorum* strain Ep-1PN was co-infected by Sclerotinia debilitation-associated RNA virus (SsDRV) and Sclerotinia sclerotiorum RNA virus L (SsRV-L), and the SsDRV is major factor responsible for the hypovirulent phenotype (Jiang et al. 2013). Zhang et al. reported the interaction of two RNA viruses, Yado-nushi virus 1 (YnV1) and Yado-kari virus 1 (YkV1), in the phytopathogenic fungus, *Rosellinia necatrix* (Zhang et al. 2016). Furthermore, a *Fusarium poae* strain MAFF 240374 was infected by 16 mycoviruses, including dsRNA, + ssRNA, and −ssRNA viruses (Osaki et al. 2016). In mycoviral co-infection, the synergistic mechanism between multiple viruses was revealed. Co-infection of the Lentinula edodes mycovirus HKB with the Lentinula edodes partitivirus 1 (LePV1) can enhance the accumulation of the LePV1 viral RNA-dependent RNA polymerase (RdRP) (Guo et al. 2017). In contrast, antagonistic mechanisms have also been identified in fungi, when Rosellinia necatrix victorivirus 1 (RnVV1) was co-infected with Mycoreovirus 1 (MyRV1) in *C. parasitica*, MyRV1 induced the up-regulated expression of the key genes for RNA silencing, *DCL2* and *AGO2*, which activated the RNAi response of the fungal host, inhibiting the replication of RnVV1 (Eusebio Cope and Suzuki 2015). In a previous study, we found that co-infection was prevalent in 182 strains of *U. virens* collected from Hainan Province, China revealed by metatranscriptomic sequencing (He et al. 2022). In particular, *U. virens* strain 287 was co-infected by nine different mycoviruses and showed the hypovirulent characteristics (He et al. 2022). The study of co-infecting pathogenic fungi has enabled exploration of viral evolution from a different perspective and identified the main factors responsible for the hypovirulent characteristics.

In this study, we found that a hypovirulent *U. virens* strain Uv325 was co-infected by four novel mycoviruses from three lineages, designated Ustilaginoidea virens RNA virus 16 (UvRV16), Ustilaginoidea virens botourmiavirus virus 8 (UvBV8), Ustilaginoidea virens botourmiavirus virus 9 (UvBV9), and Ustilaginoidea virens narnavirus virus 13 (UvNV13), respectively. Moreover, we have established an efficient and stable system of viral horizontal transmission to obtain isogenic strains infected by different mycoviruses. Importantly, we showed that UvRV16 is the major factor responsible for the hypovirulent characteristics of *U. virens* strain Uv325, resulting in slowed growth rates, reduced conidial yield and attenuated pigmentation by suppressing the transcription expression of multiple genes involved in growth, conidiation and pathogenicity. Furthermore, UvRV16 also alleviated the toxic effects of *U. virens* mycotoxins on rice seed germination and seedling growth. We elucidated a mechanism that UvRV16 RdRP can interact with UvATG6 and suppress the transcriptional expression of autophagy and RNA silencing-related genes to disrupt the antiviral response of *U. virens*. Therefore, we conclude that UvRV16 is not only a great material for studying the interactions between mycoviruses and host fungi, but also a potential biocontrol resource for control of RFS.

## Results

### *U. virens* Strain Uv325 is Coinfected by Four Potentially Novel Mycoviruses

**Fig. 1:**
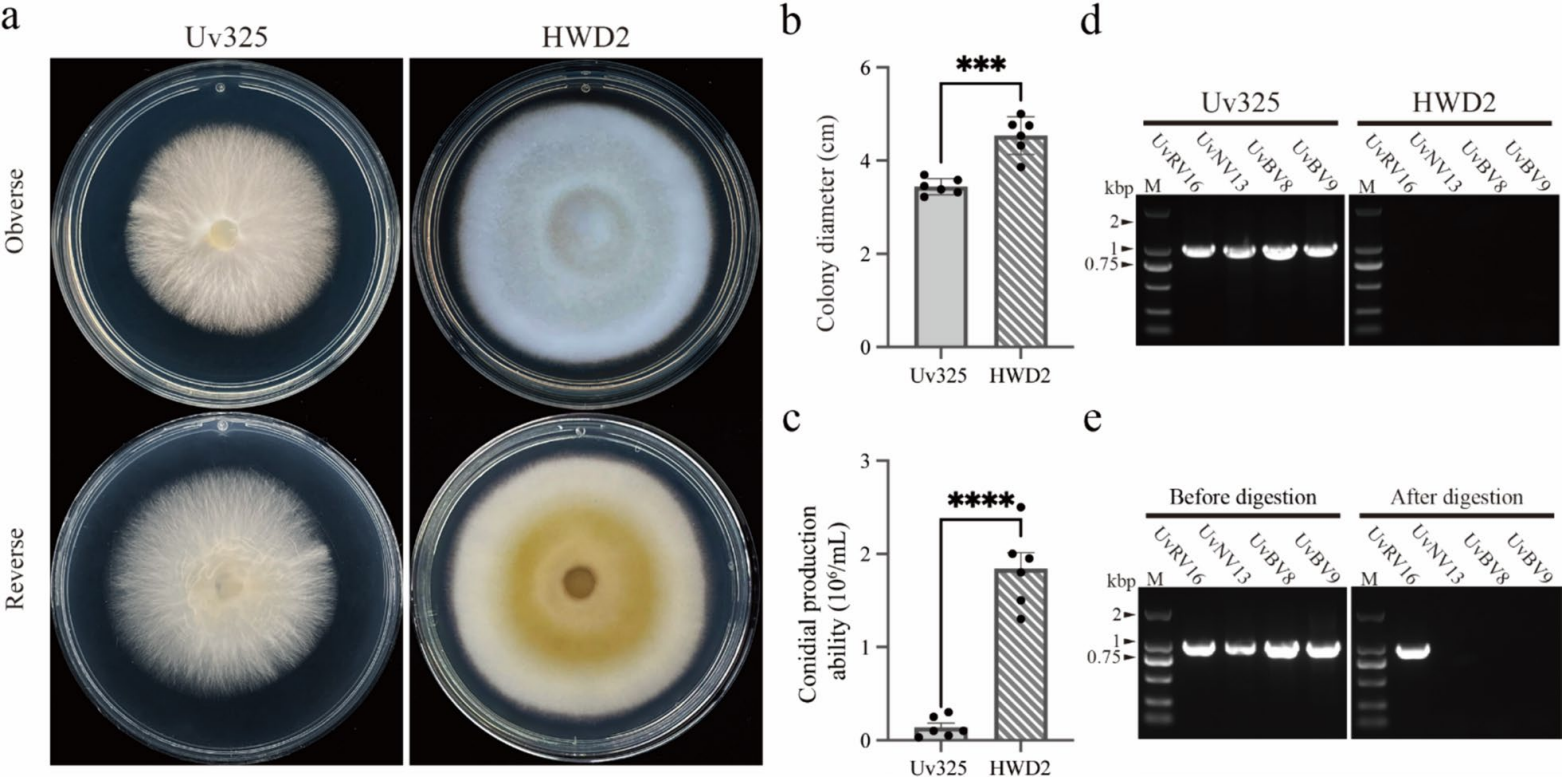
Biological characteristics of the *U. virens* strains Uv325 and HWD2. **a** Abnormal colony morphology of virus-infected strain Uv325 and virulent strain HWD2 on PSA plates after 14 days of growth in the dark at 28 °C. **b** Comparison of colony diameters of strains Uv325 and HWD2 on PSA plates after 14 days of growth in the dark at 28 °C. **c** Comparison of the conidial production ability of strains Uv325 and HWD2 after 7 days of shaking culture in the dark at 28 °C in PS liquid medium. **d** RT-PCR of total RNAs with mycovirus-specific primers based on metatranscriptomic data. **e** RT-PCR of dsRNA with mycovirus-specific primers before and after deoxyribonuclease I (DNase I) (digestion of ssDNA and dsDNA) and S1 nuclease (digestion of ssDNA or ssRNA) treatments. Lane M: DL2000 DNA molecular weight marker. The error line indicates the calibration of the mean of the three samples. Different letters at the top of each bar indicate significant differences at *P* < 0.05 confidence level according to t-test

In our previous research (He et al. 2022), a particular *Ustilaginoidea virens* strain Uv325 (Uv325) attracted our attention by its hypovirulent characteristics. Compared to virulent strain HWD2, strain Uv325 showed abnormal colony morphology, significantly slower growth, reduced conidial production and attenuated pigmentation on potato sucrose agar (PSA) (Fig. 1a-c). Therefore, we speculate that these abnormal phenotypes may be caused by mycoviral infections. We designed specific primers for reverse-transcription PCR (RT-PCR) for the detection of potential mycoviruses in strain Uv325 based on previous metatranscriptomic sequencing data (Table S1) (He et al. 2022). The results demonstrated that strain Uv325 is co-infected by at least four potentially novel mycoviruses which were not detected in virulent strain HWD2 (Fig. 1d).

To confirm the genomic characterization of these four potentially novel mycoviruses, we extracted the viral nucleic acids of strain Uv325 by CF-11 cellulose and treated with different nucleases for RT-PCR. After treatment with deoxyribonuclease I (DNase I) (digestion of ssDNA and dsDNA) and S1 nuclease (digestion of ssDNA or ssRNA), RT-PCR was still able to detect one virus, named Ustilaginoidea virens RNA virus 16 (UvRV16), and the other three undetectable viruses were named Ustilaginoidea virens narnavirus virus 13 (UvNV13), Ustilaginoidea virens botourmiavirus virus 8 (UvBV8) and Ustilaginoidea virens botourmiavirus virus 9 (UvBV9), respectively (Fig. 1e). This indicates that UvRV16 viral nucleic acids are dsRNA molecules, while UvNV13, UvBV8 and UvBV9 viral nucleic acids are + ssRNA molecules. Taken together, these results suggest that the hypovirulent phenotype of *U. virens* strain Uv325 may be due to the novel mycoviruses co-infection.

### Genome Organization of Four Novel Mycoviruses in *U. virens* Strain Uv325

We characterized the genomic organizations of UvRV16, UvNV13, UvBV8 and UvBV9 by combining metatranscriptomic sequencing data and RNA ligase-mediated (RLM)-rapid amplification of cDNA ends (RACE).

**Fig. 2:**
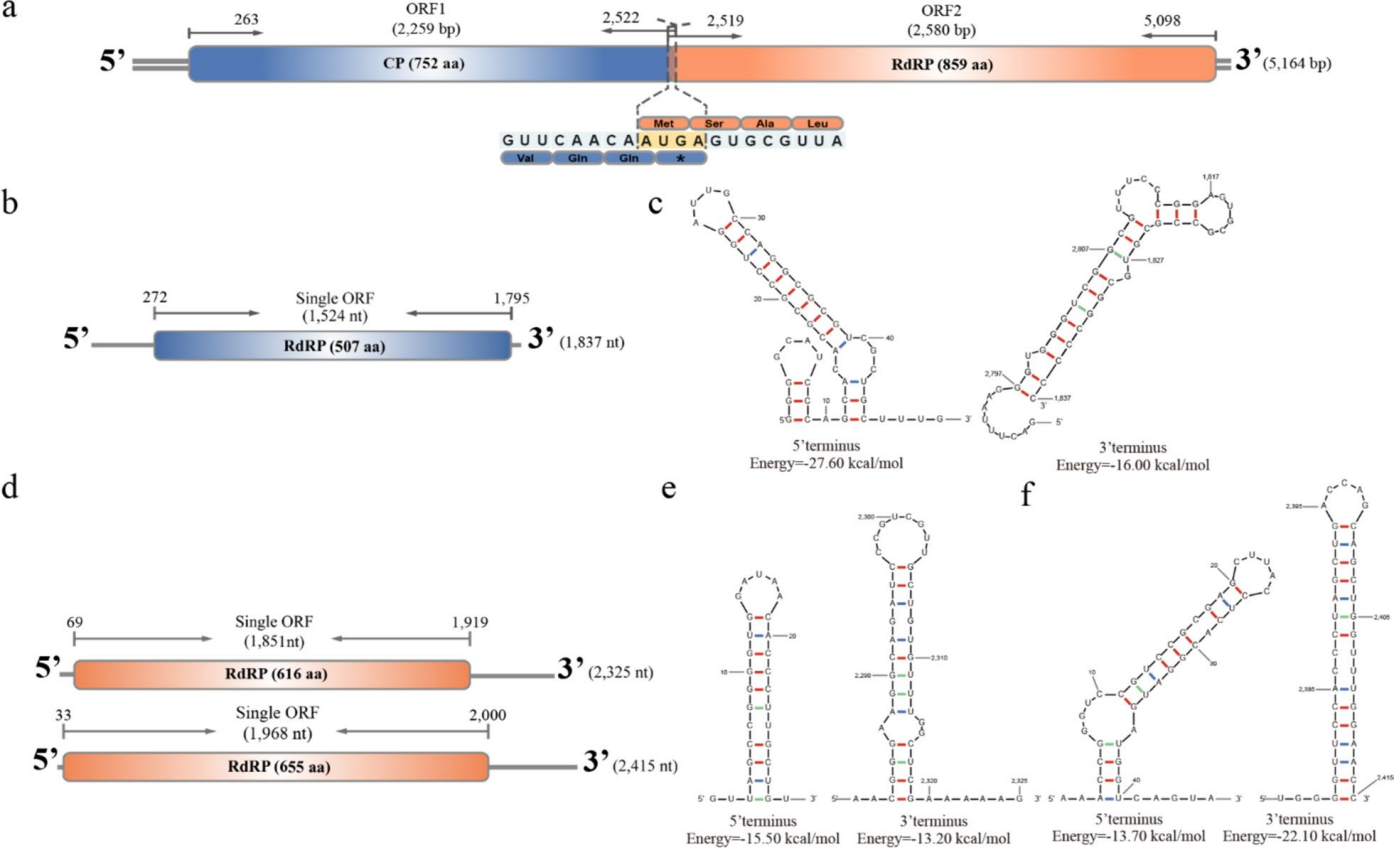
Genome organizations of four mycoviruses UvRV16, UvNV13, UvBV8 and UvBV9. **a** Schematic genome organization of UvRV16. Bolded boxes indicate the ORFs encoded by UvRV16, and UTRs as gray lines. Representative tetranucleotides (AUGA) are annotated in the schematic. Schematic of genome organizations of UvNV13, UvBV8 and UvBV9 genomes are shown in **b** and **d**, respectively. Bold boxes indicate the ORFs encoded by mycoviruses, and gray lines indicate UTRs. **c** The predicted secondary structure of the 5’-UTR (nucleotides 1–271) and 3’-UTR (nucleotides 1795–1837) of UvNV13. **e** The predicted secondary structures of the 5’-UTR (nucleotides 1–30) and 3’-UTR (nucleotides 2280–2325) of UvBV8. **f** The predicted secondary structures of the 5’-UTR (nucleotides 1–45) and 3’-UTR (nucleotides 2375–2415) of UvBV9

The complete genome of UvRV16 is 5164 bp in length containing two open reading frames (ORFs), ORF1 and ORF2. The tetranucleotide sequence (AUGA) observed in the overlap of the two ORFs, which is typical of members of the genus *Victorivirus* in the family *Totiviridae*, is the ORF2 start codon (AUG) and the ORF1 termination codon (UGA) (King et al. 2012; da Silva Camargo et al. 2023). The ORF1 (2259 bp) encodes a capsid protein (CP) with 752 amino acids (aa) that share 44.94% identity with the CP of Helminthosporium victoriae virus 190S (HvV190S, accession number O57043.2). The ORF2 (2580 bp) encodes a viral RNA-dependent RNA polymerase (RdRP) with 859 aa, which is 40.88% identical to the RdRP of Helminthosporium victoriae virus 190S (HvV190S, accession number O57044.2). The coding region is respectively flanked by 5’ and 3’ untranslated regions (UTRs) with lengths of 263 and 66 bp, respectively (Fig. 2a).

The full-length cDNA sequence of UvNV13 is 1837 nt containing one large ORF and possessing short terminal inverted repeats (5’-GGGGC and GCCCC-3’), which are similar to the terminal characteristics of Saccharomyces 23S RNA narnavirus (ScNV-23S) and Saccharomyces 20S RNA narnavirus (ScNV-20S) (Mardanov et al. 2020). The ORF (1524 nt) encodes a RdRP with 507 aa that share 28.09% identity with the ScNV-23S (accession number Q07048.2) and share 25.28% identity with the ScNV-20S (accession number P25328.1) (Fig. 2b). The predicted terminal stem-loop secondary structures showed that the 5’ and 3’ UTRs of UvNV13 could be folded into terminal stable stem-loop structures with ∆G values of −27.60 and −16.00 kcal/mol, respectively (Fig. 2c).

The genomic organizations of UvBV8 and UvBV9 are 2325 and 2415 nt, respectively, and both encode only one large ORF. The ORF of UvBV8 is 1851 nt and encodes a polyprotein with 616 aa, whereas the ORF of UvBV9 is 1968 nt and encodes a polyprotein with 655 aa. However, no similarity viral proteins were found by BLASTp alignment. We performed a BLASTn search with the viral complete genome sequence and found that UvBV8 shared 77.16% identity with the genomic sequence of Pecols virus (PV, accession number MW434079.1), while UvBV9 shared 72.56% identity with the putative RdRP of *Magoulivirus* sp. (ID: MZ679378.1). Therefore, we speculated that both ORFs of UvBV8 and UvBV9 could encode RdRP, respectively (Fig. 2d). In addition, the predicted terminal stem-loop secondary structures showed that the 5’ and 3’ UTRs of UvBV8 could be folded into terminal stable stem-loop structures with ∆G values of −15.50 and −13.20 kcal/mol, respectively (Fig. 2e). The 5’ and 3’ UTRs of UvBV9 could also be folded into terminal stable stem-loop structures with ∆G values of −13.70 and −22.10 kcal/mol, respectively (Fig. 2f).

### Sequence Alignment and Phylogenetic Analyses of Four Potentially Novel Mycoviruses

**Fig. 3:**
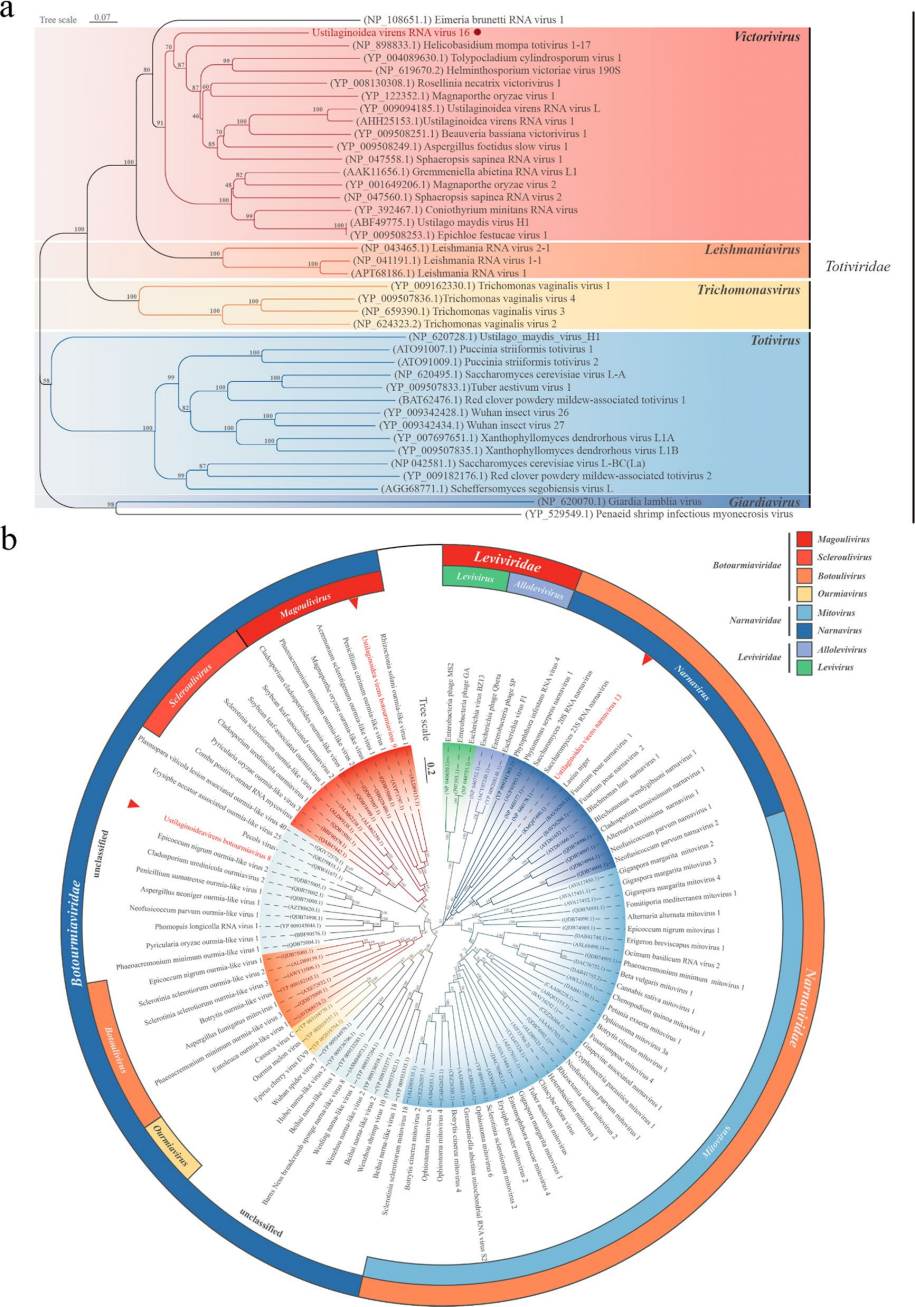
Phylogenetic analyses of four potentially novel mycoviruses. **a** Phylogenetic tree constructed from the RdRP amino acid sequences of UvRV16 and other viruses in different genus of family *Totiviridae*, the UvRV16 highlighted with black dot. **b** UvNV13, UvBV8 and UvBV9 and other viruses in different genus of families *Narnaviridae*, *Botourmiaviridae* and *Leviviridae.* The UvNV13, UvBV8 and UvBV9 highlighted in red with special symbols. Constructed by maximum likelihood (ML) using MEGA 11 software, each branch support was estimated based on 1000 bootstrap repetitions, and bootstrap values (%) were labeled on the branches

To clarify the evolutionary relationship between UvRV16 and other mycoviruses, we performed amino acid sequence alignment and phylogenetic classification based on its RdRP protein sequence. Phylogenetic tree was constructed by aligning the RdRP amino acid sequences of UvRV16 with five different viral genera (*Victorivirus*, *Leishmaniavirus*, *Trichomonasvirus*, *Totivirus* and *Giardiavirus*) and unclassified viruses in the family *Totiviridae* (Fig. 3a) (King et al. 2012; da Silva Camargo et al. 2023). The results indicated that UvRV16 formed an independent branch in the genus *Victorivirus* (Fig. 3a).

Multiple alignment based on the RdRP amino acid sequences of UvRV16 and selected viruses revealed that eight conserved motifs specific to the family *Tortiviridae* were identified from UvRV16, in addition to the generally conserved GDD motifs (Fig. S1a). Together with the results of overlapping tetranucleotide characterization (AUGA) (Fig. 2a) (Bruenn 1993; Campo et al. 2016; Kartali et al. 2019), multiple alignment and phylogenetic tree, we determined that UvRV16 is a novel mycovirus belonging to the genus *Victorivirus* in the family *Totiviridae*.

We have selected the RdRP amino acid sequences of UvBV8, UvBV9, UvNV13 and other 95 mycoviruses from eight genera in three families (*Leviviridae*, *Narnaviridae* and *Botourmiaviridae*) for multiple alignment and phylogenetic classification to clarify the evolutionary relationships of the three single-stranded mycoviruses. Phylogenetic analyses showed that UvBV8 clustered with an unclassified virus, Pecols virus, in the family *Botourmiaviridae*, UvBV9 clustered with the members in the genus *Magoulivirus* and UvNV13 clustered with ScNV-23S and ScNV-20S in the genus *Narnavirus* (Fig. 3b)*.* Multiple alignment showed that the RdRP of UvNV13 is predicted to encode eight conserved motifs unique to the members of the family *Nanaviridae* (Fig. S1b) (Lin et al. 2020; Fu et al. 2023). Similarly, multiple alignment results showed that eight conserved motifs unique to the family *Botourmiaviridae* were found in the RdRPs of UvBV8 and UvBV9 (Fig. S1c) (Liu et al. 2020). These RdRPs all contain a highly conserved typical motif “GDD” (motif IV), which is commonly found in + ssRNA mycoviruses and located at the catalytic site of the enzyme to play key role in metal binding (Vázquez et al. 2000; Zhou et al. 2023; O’Reilly and Kao 1998; Argos 1988). Taken together, these results suggest that both UvBV8 and UvBV9 are novel mycoviruses belonging to family *Botourmiaviridae*, and that UvNV13 is a new member of the genus *Narnavirus* in the family *Nanaviridae*.

### The Novel Totivirus UvRV16 is a Main Hypovirulent Factor in *U. virens*

**Fig. 4:**
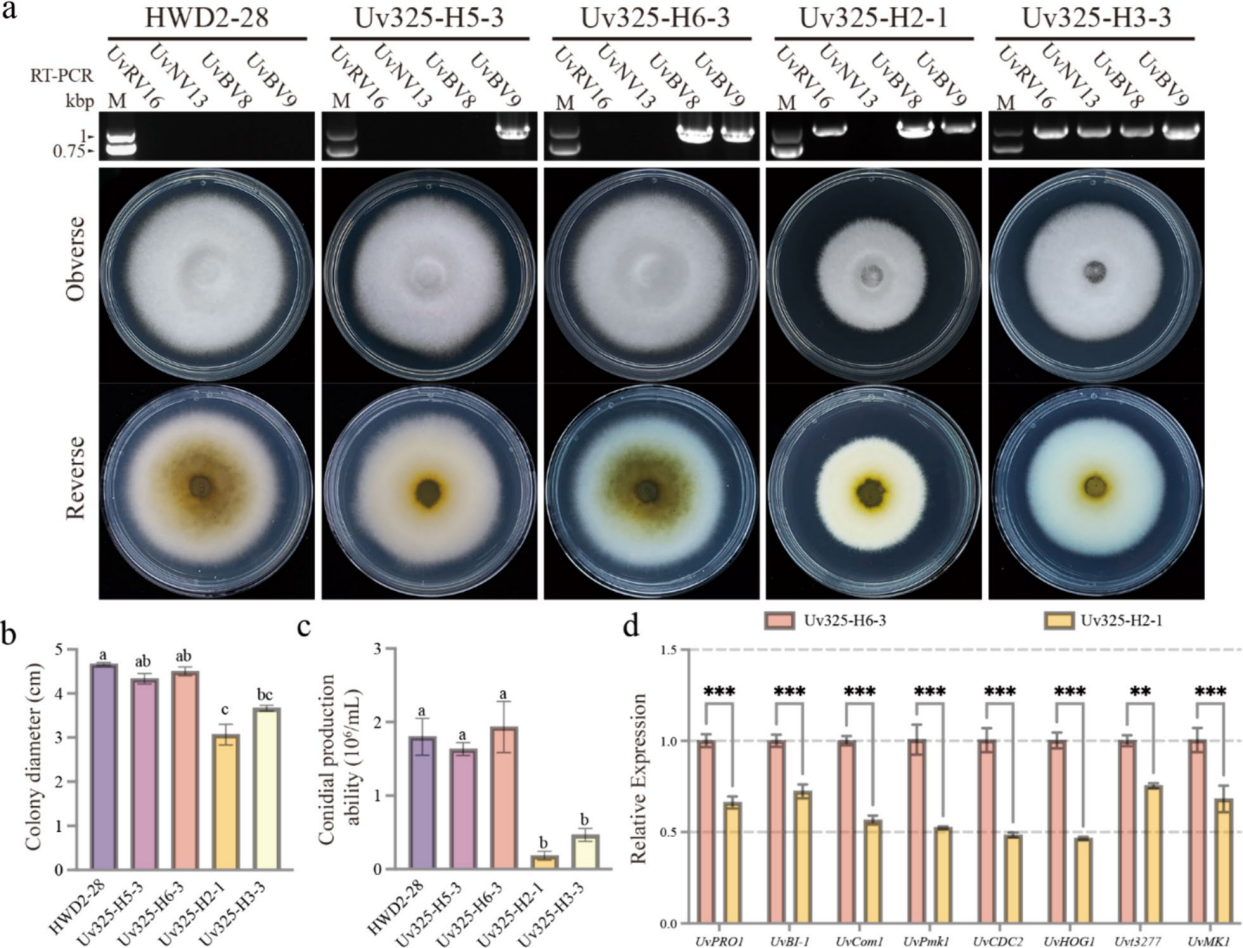
Biological characteristics of *U. virens* strain HWD2-28 and its four isogenic strains. **a** Colony morphology of *U. virens* strain HWD2-28 and its four isogenic strains cultured on PSA plates at 28 °C for 14 days in the dark. RT-PCR of total RNAs with mycovirus-specific primers. **b** Comparison of colony diameters of *U. virens* strain HWD2-28 and its four isogenic strains on PSA plates after 14 days of growth in the dark at 28 °C. **c** Comparison of the conidial yield of *U. virens* strain HWD2-28 and its four isogenic strains after 7 days of shaking culture in the dark at 28 °C in PS liquid medium. **d** Relative expression levels of conidiation-related genes as shown by qRT-PCR. Different letters at the top of each column show significant differences at the *P* < 0.05 confidence level according to the t-test

To verify the relationship between four novel mycoviruses and hypovirulence in *U. virens*, we attempted to obtain isogenic strains infected by one or several viruses using dual-cultures. *U. virens* strain HWD2-28 with hygromycin B resistance was used as a recipient strain for mycoviruses transmission and it was dual-cultured with hypovirulent *U. virens* strain Uv325, which was infected with four novel mycoviruses. Strains Uv325 and HWD2-28 were dual-cultured in the same PSA plates, and after 14 days, the mycelial plugs from three different locations were transferred to PSA plates supplemented with hygromycin B and cultured three generations for screening (Fig. S2). Fortunately, we obtained four isogenic strains infected with different viruses by dual-cultures, named strain Uv325-H2-1, strain Uv325-H3-3, strain Uv325-H5-3, and strain Uv325-H6-3. The mycovirus-specific RT-PCR results showed that strain Uv325-H5-3 was infected only by UvBV9 (Fig. 4a). In addition, strain Uv325-H6-3 was co-infected by viruses UvBV8 and UvBV9, and strain Uv325-H2-1 was co-infected by viruses UvRV16, UvBV8 and UvBV9 (Fig. 4a). Furthermore, strain Uv325-H3-3 was co-infected by four novel mycoviruses (Fig. 4a). In addition, to investigate potential vertical transmission of these four mycoviruses in *U. virens* strain Uv325, we isolated individual conidia from mycelium and cultured on PSA. A total of 20 single subisolates were randomly selected and analyzed for the presence of the four mycoviruses. The experiments showed that all subisolates were infected with all four mycoviruses, confirming effective vertical transmission of these viruses (Fig. S3).

To explore the biology of the novel mycovirus, we evaluated the colony morphology, growth rate and conidial yield between the four isogenic strains and the control strain HWD2-28. In terms of colony morphology, after 14 days of cultivation in PSA medium, the pigmentation of strains Uv325-H2-1, Uv325-H3-3, and Uv325-H5-3 were significantly attenuated compared to the strain HWD2-28, which is the main characteristic of hypovirulent (Fig. 4a). Comparison of colony diameters after culturing at 14 days, we found that a significant decrease in the growth rates of strains Uv325-H2-1 and Uv325-H3-3 compared to strain HWD2-28. These two isogenic strains were characterized by all being infected with UvRV16 (Fig. 4a-c). The WT strain had formed 1.8 × 10^6^ conidia/mL after 7 days of culture, whereas strain Uv325-H5-3 and strain Uv325-H6-3 had produced 1.6 × 10^6^ conidia/mL and 1.9 × 10^6^ conidia/mL, respectively, under the same conditions, with no significant difference. In contrast, strain Uv325-H2-1 and strain Uv325-H3-3 had formed 0.18 × 10^6^ conidia/mL and 0.47 × 10^6^ conidia/mL, respectively, after UvRV16 infection, which were significantly decreased compared to the WT strain (Fig. 4c). The expression of eight genes involved in conidiation of filamentous fungi were significantly downregulated in UvRV16-infected strains, including C6 zinc fnger transcription factors *UvPRO1*, Bax inhibitor-1 (BI-1) protein *UvBI-1*, transcription factor *UvCom1*, CMGC kinase *UvPmk1* and *UvCDC2*, the ortholog of yeast HOG1 MAP kinase *UvHog1*, homology with low-affinity iron transporter protein Uvt3277, homology with mitogen-activated protein kinase UvMK1 (Fig. 4d). Interestingly, we found that the co-infection with UvNV13 could weaken in some degree the effect of UvRV16 on the growth rate and conidial production of *U. virens* by comparing Uv325-H2-1 and Uv325-H3-3. Taken together, UvRV16 infection resulted in slowed growth rate, reduced conidial yield, and attenuated pigmentation in *U. virens* strain HWD2-28. We thereby confirmed that the novel totivirus, UvRV16, is the major factor responsible for the hypovirulent phenotype of *U. virens*.

### UvRV16 Reduces Inhibitory Effects of *U. virens* Mycotoxins on Rice Seed Germination and Seedling Growth

Ustilaginoidins are one of the major types of mycotoxins produced by *U. virens*, some ustilaginoidins involved in the pathogenesis as virulence factors and were able to interfere the physiological functions of the rice cells (Chen et al. 2021; Zhang et al. 2022; Wen et al. 2023). Studies have shown that Ustilaginoidins O, E, F, R2, B, I and U play important roles in inhibiting rice seedling growth (Lu et al. 2015).

**Fig. 5:**
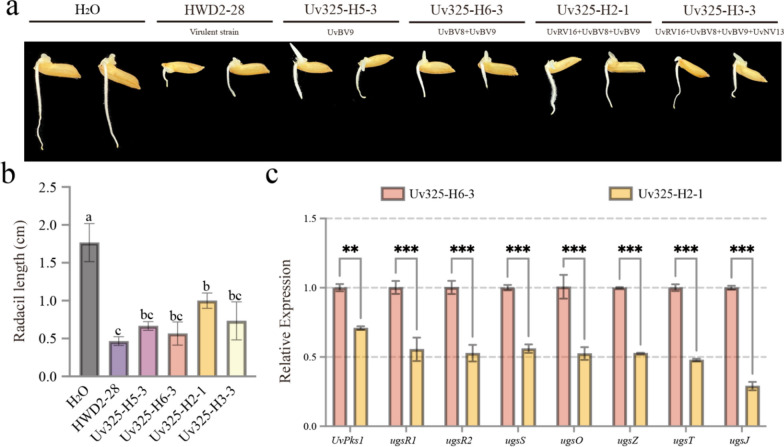
UvRV16 interfered the expression of toxin-related genes and reduced the inhibitory effects of *U. virens* mycotoxins on rice seed germination. **a**-**b** The inhibitory effect of mycotoxins of *U. virens* strain HWD2-28 and four isogenic strains on rice seed germination. Rice seeds were soaked in crude mycotoxins for 2 days and root length was determined. Seeds treated with sterile distilled water were used as control (CK). These experiments were repeated three times with 20 seeds each time. Different letters at the top of each column show significant differences at the *P* < 0.05 confidence level according to the t-test. **c** The relative expression levels of eight *U. virens* mycotoxins synthesis-related genes were verification by qRT-PCR analyses

To detect the effects of mycoviruses on the production of mycotoxins in *U. virens*, we extracted the *U. virens* crude mycotoxins from PS liquid medium cultures of strains HWD2-28, Uv325-H5-3, Uv325-H6-3, Uv325-H2-1, and Uv325-H3-3, respectively. Rice seeds were treated separately with the crude mycotoxins extracted from the cultures of *U. virens* strains to determine whether mycoviruses influence the inhibitory effects of mycotoxin compounds on rice seed germination. As shown in Fig. 5a-b, the rice shoots treated with the crude mycotoxins extracted from strain Uv325-H2-1 (co-infected of UvRV16, UvBV8 and UvBV9) were significantly longer than those in other treatments. Whereas the rice shoots treated with the crude mycotoxins extracted from strain Uv325-H6-3 (co-infected of UvBV8 and UvBV9) were shorter those treated with the crude mycotoxins extracted from strain Uv325-H2-1 (co-infected of UvRV16, UvBV8 and UvBV9), suggesting that UvRV16 is a major factor in alleviating the inhibitory effects of mycotoxins on rice seed germination. However, the rice shoots treated with the crude mycotoxins extracted from strain Uv325-H3-3 (co-infected by UvRV16, UvBV8, UvBV9 and UvNV13) were also shorter, suggesting that co-infection with UvNV13 may weaken the inhibitory effect of UvRV16 in some degree. The synthesis gene cluster of ustilaginoidins has been demonstrated to be critical for mycotoxin biosynthesis and modifcations (Li et al. 2019), including the polyketide synthase UvPKS1, transcription initiation factor ugsR1, transcription factor ugsR2, hypothetical protein ugsS and ugsZ, FAD binding domain protein ugsO, major facilitator superfamily transporter ugsT and methyltransferase ugsJ. We found that 8 genes involved in the biosynthesis of mycotoxins in *U. virens* were significantly down-regulated in strain Uv325-H2-1 (co-infected of UvRV16, UvBV8 and UvBV9) compared to strain Uv325-H6-3 (co-infected of UvBV8 and UvBV9) by qRT-PCR (Fig. 5c).

**Fig. 6:**
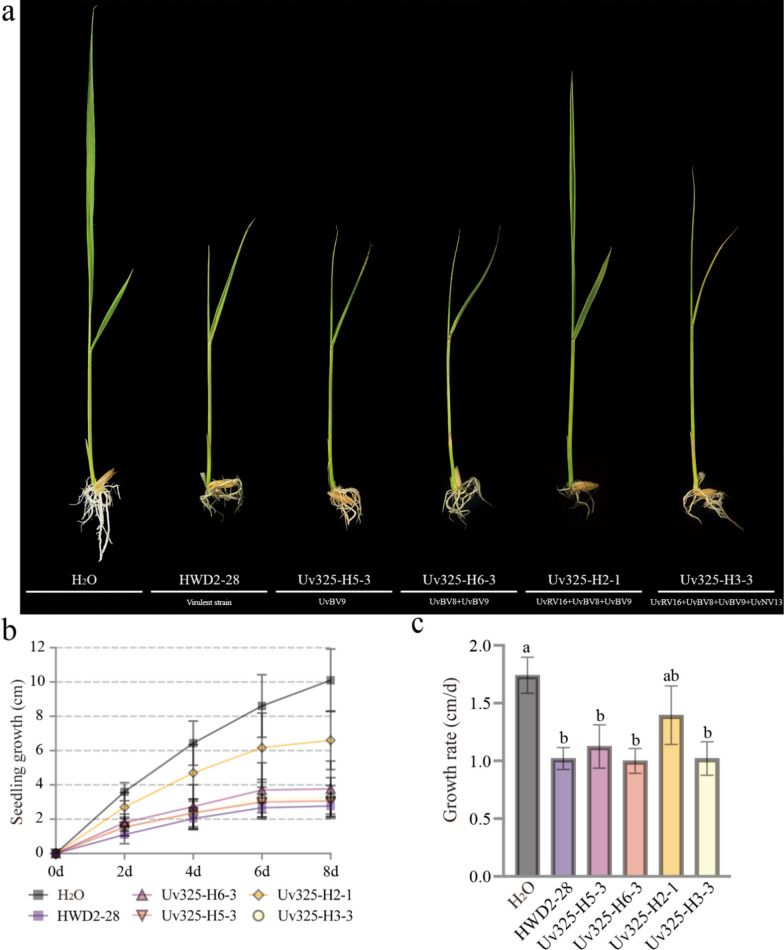
UvRV16 significantly suppressed the inhibitory effects of *U. virens* mycotoxins on rice seedling growth. **a** The morphology of rice seedlings treated with mycotoxins. The roots of equal-height rice seedlings were soaked in 15 mL-tube containing 5 mL crude mycotoxins, and the height of rice seedlings were measured after 8 days. These experiments were repeated three times at 28 °C under 12 h light/12 h dark. Different letters at the top of each column show significant differences at the *P* < 0.05 confidence level according to the t-test. **b** The length of rice seedlings at 2, 4, 6 and 8 days after mycotoxin treatment. **c** The average growth rates of rice seedlings during 8 days after mycotoxin treatment

Furthermore, rice seedlings were treated separately with the crude mycotoxins extracted from the cultures of *U. virens* strains to explore whether mycoviruses influence the inhibitory effects of mycotoxin compounds on rice seedling growth. We measured the lengths of rice seedlings at 2, 4, 6, and 8 days after mycotoxin treatment (Fig. 6b) and calculated the average growth rates of rice seedlings during 8 days after mycotoxin treatment (Fig. 6c) to evaluate the effect of mycoviruses on mycotoxin production. By comparing the growth dynamics and average growth rates of rice seedlings, we found that crude mycotoxins extracted from strain Uv325-H2-1 (co-infected with UvRV16, UvBV8 and UvBV9) significantly inhibited the growth of rice seedlings, whereas crude mycotoxins extracted from strain Uv325-H6-3 (co-infected with UvBV8 and UvBV9) were not significantly inhibited rice seedling growth. (Fig. 6). Interestingly, the inhibitory effects of the crude mycotoxins extracted from strain Uv325-H3-3 (co-infected by UvRV16, UvBV8, UvBV9 and UvNV13) were also not significant (Fig. 6). These results suggest that UvRV16 is the major factor responsible for attenuating the inhibitory effects of mycotoxins extracted from the cultures of *U. virens* on rice seedling growth, whereas co-infection with UvNV13 of *U. virens* strains can weaken the effects of UvRV16 on mycotoxin production in some degree. Taken together, these studies suggest that UvRV16 can suppress the expression of mycotoxin biosynthetic genes to alleviate the inhibitory effect of *U. virens* crude mycotoxins on rice seed germination and seedling growth.

### UvRV16 Significantly Inhibits the Antiviral Response of *U. virens*

**Fig. 7:**
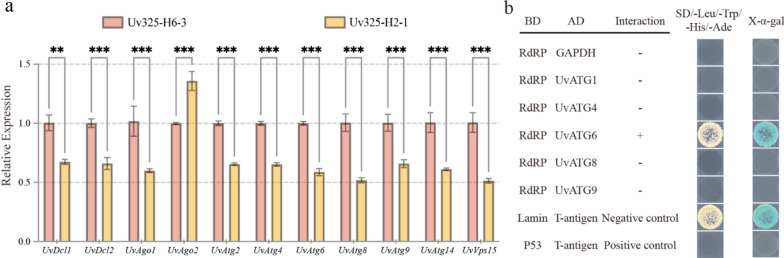
UvRV16 significantly inhibited the antiviral response of *U. virens*. **a** qRT-PCR analysis of the transcript levels of autophagy and RNAi-related genes in strain Uv325-H6-3 (infected with UvBV8 and UvBV9) and strain Uv325-H2-1 (infected with UvRV16, UvBV8 and UvBV9). **b** Yeast two-hybrid (Y2H) assays for possible interactions between UvRV16 RdRP and autophagy-related protein 6 in *U. virens* (UvATG6)

The important role of autophagy and RNA silencing (RNAi) in plant defense response against plant viruses has been demonstrated (Yang et al. 2023; Huang et al. 2023; Liu et al. 2023), yet their roles in the interactions between mycoviruses and fungal hosts are still unknown. To investigate whether UvRV16 infection regulates antiviral response in *U. virens*, we first examined the expression of autophagy and RNAi-related genes in strain Uv325-H6-3 (infected with UvBV8 and UvBV9) and strain Uv325-H2-1 (infected with UvRV16, UvBV8 and UvBV9) using qRT-PCR. The results showed that three RNAi-related genes (*UvDcl1*, *UvDcl2* and *UvAgo1*) and seven autophagy-related genes (*UvAtg2*, *UvAtg4*, *UvAtg6*, *UvAtg8*, *UvAtg9*, *UvAtg14*, and *UvVps15*) were significantly down-regulated, with the exception of one RNAi-related gene (*UvAgo2*) which was significantly up-regulated (Fig. 7a), suggesting that UvRV16 infection may suppress host antiviral response. To investigate the mechanism underlying the suppression of autophagy in *U. virens* by UvRV16, we screened the host factors that interact with UvRV16 through Y2H assay. The results of Y2H assays indicated that the positive protein–protein interaction was found between UvRV16 RdRP and UvATG6, whereas no interaction was evident between UvRV16 RdRP with any other autophagy-related proteins (Fig. 7b). Together, these results suggest that UvRV16 may inhibit *U. virens* autophagy by interacting with UvATG6, resulting to the promotion of the replication and infection of UvRV16 in *U. virens*.

## Discussion

Viruses of the family *Totiviridae* are dsRNA viruses and the genome contains two large, usually overlapping ORFs that encode RdRP and CP, respectively (Shi et al. 2023). Members of the family *Nanaviridae* are the simplest genomes and encoding only a single RdRP (Hillman and Cai 2013; Espino Vázquez et al. 2020). The family *Botourmiaviridae* is a newly established taxonomic taxon in recent years that includes viruses with positive-sense RNA genomes infecting plants and fungi (Ayllón et al. 2020). Here, we identified the co-infection of four novel mycoviruses in a hypovirulence-associated *U. virens* strain Uv325, named Ustilaginoidea virens RNA virus 16 (UvRV16), Ustilaginoidea virens narnavirus 13 (UvNV13), Ustilaginoidea virens botourmiavirus 8 (UvBV8) and Ustilaginoidea virens botourmiavirus 9 (UvBV9), respectively. The complete genome of UvRV16 is 5164 bp in size and contains two ORFs encoding RdRP and CP, respectively. It is a novel mycovirus belonging to the genus *Victorivirus* in the family *Totiviridae*. The UvNV13 is a novel mycovirus belonging to the genus *Narnavirus* in the family *Nanaviridae* that is 2302 nt in size and contains a large ORF encoding RdRP. The genomic organizations of UvBV8 and UvBV9 are 2325 and 2415 nt, respectively, and both contain a large ORF encoding RdRP, which are new members belonging to the family *Botourmiaviridae*. These results suggest that *U. virens* strain Uv325 harbors a complex and diverse mycovirus community. This research expands our knowledge of the diversity and evolution of viruses in the families *Totiviridae*, *Nanaviridae*, and *Botourmiaviridae*.

Co-infection by two or more mycoviruses is very common among all major taxa of fungi. Jiang et al. reported that *U. virens* strain GX-1 was co-infected by four mycoviruses, including Ustilaginoidea virens RNA virus 1 (UvRV1) belonging to the family *Totiviridae*, the unclassified Ustilaginoidea virens RNA virus 4 (UvRV4) and two viruses belonging to the family *Partitiviridae* (Jiang et al. 2014). Mu et al. characterized nine mycoviruses assigned into eight possible families infecting *Sclerotinia sclerotiorum* strain SX276. Furthermore, they have applied a series of strategies to eliminate one or more viruses in strain SX276 to verify the relationship between mycoviruses and hypovirulence in *S. sclerotiorum* (Mu et al. 2021). Their results demonstrated that Sclerotinia sclerotiorum endornavirus 3 (SsEV3) is an important factor responsible for hypovirulent phenotypes in *S. sclerotiorum* (Mu et al. 2021). In the present study, we obtained isogenic strains infected with different viruses by dual culture to explore the main factors responsible for the hypervirulent phenotype. Compared with the control strain HWD2-28, the growth rates and conidial yields of strains Uv325-H2-1 and Uv325-H3-3 infected with UvRV16 were significantly reduced, whereas the phenotypes of strain Uv325-H6-3 infected with both UvBV8 and UvBV9 were not significantly altered. Interestingly, we found that co-infection with UvNV13 could attenuate in some degree the hypovirulence induced by UvRV16. Based on the qRT-PCR results we hypothesized that UvRV16 may have decreased conidial yield by suppressing the expression of conidiation-related genes in *U. virens*. This is the first report that identification of mycoviruses with hypovirulent characteristics in *U. virens*. Therefore, we considered that UvRV16 is a good biocontrol factor for the control of RFS.

False smut balls formed during *U. virens* infection of rice spikes contain multiple types of mycotoxins, including ustiloxins and ustilaginoidins, which are serious threats to human and animal health (Li et al. 2019). Although the role of mycotoxins in *U. virens* infection is largely unknown, there are biochemical studies strongly confirming that some pathogenic genes are responsible for the biosynthesis pathway of mycotoxins (Li et al. 2019; Sun et al. 2020; Yang et al. 2023). For example, UvPKS1 was proven to be a key enzyme responsible for the biosynthesis of ustilaginoidin protein (Li et al. 2019). UvHOG1 is not only important for mycelial growth and conidial yield, but also is involved in regulating the production of phytotoxic compounds, and the Uvhog1 mutant reduced the inhibitory properties of mycotoxins on rice shoots (Zheng et al. 2016). Wen et al*.* reported that AgNPs are an effective nanofungicide against RFS, but AgNPs promote the expression of ustilaginoidin biosynthesis genes, leading to an increase of ustilaginoidins production in *U. virens* (Wen et al. 2023). Mycotoxins may act as a virulence factor during infection in order to adapt the changes in the infection environment (Zhang et al. 2014b, 2022; Li et al. 2019; Wen et al. 2023; Liu et al. 2023). In this study, we found that UvRV16 attenuated the toxic effects of *U. virens* mycotoxins on rice seed germination and seedling growth. Therefore, we showed that mycoviruses may be the more suitable biocontrol factors for controlling RFS compared to other control strategies.

In the co-evolutionary arms race between plants and viruses, many defense and counter-defense strategies have been successively established (Hafrén et al. 2018; Li et al. 2018; Wang et al. 2022). To combat viral infections, plants have evolved various complex and systemic defense mechanisms to limit viral replication and transmission. For example, host RNA silencing can target degradation of viral nucleic acids to limit replication after virus infection. Emerging evidence has demonstrated that viral infection also activates host autophagy to selectively degrade viral proteins. For plant viruses, Huang et al. propose a conserved mechanism that plant SnRK1 senses rhabdovirus glycoprotein to initiate autophagy and degrade it via ATG6 to limiting the toxicity of viral glycoprotein and restricting the infection of rhabdovirus (Huang et al. 2023). Turnip mosaic virus (TuMV), a + ssRNA virus, infection activates autophagy in plants, and the core component of autophagy, Beclin1 (ATG6), inhibits viral replication (Li et al. 2018). The capsid protein of Coconut mosaic virus (CaMV) was reported to be targeted degraded by *Arabidopsis* NBR1 and restricted the viral infection (Hafrén et al. 2017). Yang et al. reported that Barley stripe mosaic virus (BSMV) γb protein inhibits autophagy-mediated antiviral defense and promotes viral replication and infection by competitively disrupting host ATG7-ATG8 interactions (Yang et al. 2018). For human viruses, two proteins encoded by Human cytomegalovirus (HCMV), TRS1 and IRS1, which are characterized by inhibiting autophagy during infection (Mouna et al. 2016). For mycoviruses, transcription levels of *C. parasitica* genes *dcl1* and *dcl2* were significantly increased following CHV1 infection (Zhang et al. 2008). Moreover, CHV1 infection of the *dcl2* null mutant Δ*dcl2* or the *agl2* null mutant Δ*agl2* resulted in a severely debilitated growth phenotype and significantly increased virus accumulation, indicating that the genes *dcl2* and *agl2* are involved in the RNAi antiviral response (Segers et al. 2007; Sun et al. 2009). Conversely, to combat the RNAi antiviral response in *C. parasitica*, CHV1 encodes the viral suppressor of RNA silencing (VSR), p29, which promotes the accumulation of viral nucleic acids (Segers et al. 2006). In this study, we show that UvRV16 infection significantly suppressed the expression of RNAi and autophagy-related genes in *U. virens*, and the UvRV16 RdRP interacted with UvAtg6, a core component of the class III PtdIns3K complex (Huang et al. 2023). Therefore, we speculate that virus infection activates the *U. virens* antiviral pathway, resulting in degradation of viral nucleic acids via RNAi and delivery of viral proteins to autophagosomes for degradation.

## Materials and Methods

### Fungal Strains and Culture Conditions

The *U. virens* strain Uv325 was originally isolated from the diseased panicle of rice infected with *U. virens*, which were collected in Hainan province, China. The *U. virens* strain HWD2 was kindly provided by Professor Junbin Huang from Huazhong Agriculture University (Hubei province, China). The *U. virens* strain HWD2-28 is hygromycin B resistance and was used for viral transmission assays in this study. For routine cultures, *U. virens* strain was grown on potato sucrose agar (PSA, potato 200 g/L, sucrose 20 g/L, agar 17 g/L) medium at 28 °C, and liquid cultures were carried out on potato sucrose (PS, potato 200 g/L, sucrose 20 g/L) medium at 28 °C in the dark with 180 r/min. All strains were stored at 4 °C and −20 °C, respectively.

### Nucleic Acid Extraction, Molecular Cloning, Assembly and Analysis

Total RNA was extracted from 100 mg of fresh mycelia using the TransZol Up Plus RNA kit (TransGen Biotech, Beijing, China) according to the manufacturer’s instructions, and dsRNA was extracted using CF-11 cellulose (Sigma) as previously described (Herrero et al. 2012). Total RNA and dsRNA were evaluated for integrity and purity using 1% agarose gel electrophoresis and NanoDrop 2000 (Thermo Scientific, Wilmington, Del., USA), respectively. The extracted dsRNA was treated with DNase I and S1 nuclease (TaKaRa, Dalian, China) to confirm its properties. Extracted nucleic acids were reverse transcribed using TransScript One-Step gDNA Removal and cDNA Synthesis SuperMix kit (TransGen Biotech, Beijing, China).

The 5’ and 3’ terminal unknown sequences of mycoviruses were obtained by reference to a previously reported RNA ligase-mediated rapid amplification of cDNA ends (RLM-RACE) (Darissa et al. 2010). PCR amplicons were inserted into pCE2 TA/Blunt-Zero vector (Vazyme Biotech, Nanjing, China) and transformed into *Escherichia coli*, and positive clones were sequenced at least three times. The complete genome of viruses was assembled by using the DNAMAN software with default parameters. We performed sequence similarity searches by the National Center for Biotechnology Information (NCBI) Blast program (http://blast.st-va.ncbi.nlm.nih.gov/Blast.cgi). The secondary structures of the terminal of viral genomes were predicted by RNAfold Webserver (http://rna.tbi.univie.ac.at//cgi-bin/RNAWebSuite/RNAfold.cgi). To analyze open reading frames (ORFs) in mycovirus genomes, we used the NCBI ORF Finder program (https://www.ncbi.nlm.nih.gov/orffinder/). To obtain the conserved motifs, we predicted the conserved motifs using an online website (http://www.genome.jp/tools/motif/). The amino acid sequences of novel mycoviruses and similar viruses were multiple alignments by an online website (https://smart.embl.de/smart/set_mode.cgi?NORMAL=1) and displayed by SnapGene 6.0.2 software (Letunic et al. 2021). The maximum-likelihood (ML) phylogenetic trees were constructed using MEGA X with 1000 bootstraps (Walker et al. 2015), and were adjusted using the ChiPplot visualisation tool (https://www.chiplot.online/#Phylogenetic-Tree) (Xie et al. 2023).

### Viral Horizontal Transmission Assay

To obtain isogenic strains with different viruses, we co-cultured the virus-infected strain Uv325 (donor) with the virus-free strain HWD2-28 (recipient) 1 cm apart on the same PSA dish at 28 °C in dark (Fig. S2a). After culturing for 14 days, mycelial agar plugs were taken from the inner, middle and edges of each colony to obtain recipient-derived strains (Fig. S2b). All recipient-derived strains were transferred to fresh PSA plate containing 200 µg/mL of hygromycin B for three generations and analyzed for the infection of viruses via RT-PCR.

### Biological Characterization

To explore the biological effects of mycoviruses on *U. virens*, we assessed the colony morphology, mycelial growth, and conidiation. For colony morphology and mycelial growth, the agar plugs with a diameter of 5 mm were placed in the center of PSA medium for 14 days of dark at 28 °C. Subsequently, photographs were taken to record colony morphology and the colony diameter were measured using cross measurement method. For the conidial yield assays, four 5 mm diameter agar plugs were inoculated into a 250 mL flask containing 100 mL of liquid PS medium. The samples were incubated at 28 °C for 7 days at 180 r/min. Samples were filtered and used to measure conidial concentration. All of the experiments were repeated three times with three replicates each time.

### Quantitative Reverse Transcription Polymerase Chain Reaction (qRT-PCR)

To determine the effect of UvRV16 on the expression of genes related to growth, conidiation, toxins and antivirus in *U. vriens*, qRT-PCR analyses were performed. qRT-PCR reactions were carried out on a CFX96 TouchTM real-time PCR detection system (Bio-Rad, Hercules, CA, USA) using the ChamQ Universal SYBR qPCR Master Mix (Vazyme Biotech, Nanjing, China). Three independent biological replicates were conducted, each in technical triplicates. The expression level of the gene *βTtbulin* in *U. virens* was used as an internal control, and the relative expression levels were calculated by the 2^−ΔΔC(t)^ method.

### Toxicity Assays of Crude Mycotoxins of *U. virens*

First, the culture filtrates were collected from the cultures of *U. virens* after 14 days of incubation at 28 °C, 180 rpm/min for preparation of crude mycotoxins according to the previously described method (Fu et al. 2022). The 100 mL of culture filtrates were concentrated to dryness by rotary evaporator at 60 hpa and 60 °C. To adequately extract crude mycotoxins from the dry matter, 100 mL of methanol were mixed with the dry matter and shaken at 150 rpm for 2 h followed by centrifugation at 8000 rpm for 10 min to remove insoluble matter. The supernatant was concentrated to dry extract by a rotary evaporator at 60 °C under 120 hpa. Finally, the dry extract was dissolved in 100 mL of ddH_2_O to obtain crude mycotoxins and stored at 4 °C.

For the toxin inhibition of rice seed germination assay, rice seeds were incubated on filter paper soaked with 2 mL of crude mycotoxin as described previously, with 20 rice seeds treated at per time (Zhang et al. 2022). For the crude mycotoxin inhibition of rice seedling growth assays, the roots of equal-height rice seedlings were soaked in a 15 mL-tube containing 5 mL crude mycotoxin. The length of rice seed shoots was measured after 2 days of culture and the height of rice seedlings were measured after 8 days, respectively. These experiments were repeated three times at 28 °C under 12 h light/12 h dark.

### Statistical Analysis

Statistical analyses were conducted using the GraphPad Prism version 6.0 (GraphPad Software Inc., La Jolla, CA, USA), and one-way analysis of variance (ANOVA) were used to analyze the significant differences between the control and treatment groups. Significant differences occurred when *P* < 0.05.

### Supplementary Information

Additional file 1. 

Additional file 2. 

## Data Availability

All the RNA-seq data generated in this research were deposited in the Sequence Read Archive database (www.ncbi.nlm.nih.gov/sra) at NCBI under accession numbers are as follows: Ustilaginoidea virens RNA virus 16 (OR523129.1), Ustilaginoidea virens narnavirus virus 13 (OR523133.2), Ustilaginoidea virens botourmiavirus virus 8 (OR523131.1), Ustilaginoidea virens botourmiavirus virus 9 (OR523132.1).

## Abbreviations

UvRV16: Ustilaginoidea virens RNA virus 16
UvBV8: Ustilaginoidea virens botourmiavirus virus 8
UvBV9: Ustilaginoidea virens botourmiavirus virus 9
UvNV13: Ustilaginoidea virens narnavirus virus 13
ICTV: International Committee on Taxonomy of Viruses
dsRNA: Double-stranded RNA
ssRNA: Single-stranded RNA
SsHADV-1: Sclerotinia sclerotiorum hypovirulence-associated DNA virus 1
RFS: Rice false smut
DON: Deoxynivalenol
UvRV1-6: Ustilaginoidea virens RNA virus 1-6
UvPV1-4: Ustilaginoidea virens partitivirus 1-4
UvNV1-2: Ustilaginoidea virens nonsegmented virus 1-2
UvURV-HNND-1: Ustilaginoidea virens unassigned RNA virus HNND-1
SsDRV: Sclerotinia debilitation-associated RNA virus
SsRV-L: Sclerotinia sclerotiorum RNA virus L
YnV1: Yado-nushi virus 1
YkV1: Yado-kari virus 1
LePV1: Lentinula edodes partitivirus 1
RdRP: RNA-dependent RNA polymerase
RnVV1: Rosellinia necatrix victorivirus 1
MyRV1: Mycoreovirus 1
Uv325: *Ustilaginoidea virens* Strain Uv325
PSA: Potato sucrose agar
DNase I: Deoxyribonuclease I
RLM: RNA ligase-mediated
RACE: Rapid amplification of cDNA ends
ORFs: Open reading frames
CP: Capsid protein
aa: Amino acids
UTRs: Untranslated regions
ScNV-23S: Saccharomyces 23S RNA narnavirus
ScNV-20S: Saccharomyces 20S RNA narnavirus
HvV190S: Helminthosporium victoriae virus 190S
PV: Pecols virus
qRT-PCR: Quantitative real-time reverse-transcription PCR
RT-PCR: Reverse-transcription PCR
SsEV3: Sclerotinia sclerotiorum endornavirus 3
TuMV: Turnip mosaic virus
CaMV: Coconut mosaic virus
BSMV: Barley stripe mosaic virus
HCMV: Human cytomegalovirus
VSR: Viral suppressor of RNA silencing
RLM-RACE: RNA ligase-mediated rapid amplification of cDNA ends
NCBI: National Center for Biotechnology Information
ML: Maximum-likelihood
ANOVA: One-way analysis of variance

## References

[CR1] Anagnostakis SL (1982). Biological control of chestnut blight. Science.

[CR2] Argos P (1988). A sequence motif in many polymerases. Nucleic Acids Res.

[CR3] Ayllón MA, Turina M, Xie JT (2020). ICTV virus taxonomy profile: *Botourmiaviridae*. J Gen Virol.

[CR4] Bruenn JA (1993). A closely related group of RNA-dependent RNA polymerases from double-stranded RNA viruses. Nucleic Acids Res.

[CR5] Campo S, Gilbert KB, Carrington JC (2016). Small RNA-based antiviral defense in the phytopathogenic fungus *Colletotrichum higginsianum*. PLoS Pathog.

[CR6] Chen XY, Pei ZX, Li PP (2021). Quantitative proteomics analysis reveals the function of the putative ester cyclase UvEC1 in the pathogenicity of the rice false smut fungus *Ustilaginoidea virens*. Int J Mol Sci.

[CR7] da Silva Camargo M, Geremia F, Sbaraini N (2023). Molecular characterization of a novel victorivirus (order *Ghabrivirales*, family *Totiviridae*) infecting *Metarhizium anisopliae*. Arch Virol.

[CR8] Darissa O, Willingmann P, Adam G (2010). Optimized approaches for the sequence determination of double-stranded RNA templates. J Virol Methods.

[CR9] Espino Vázquez AN, Bermúdez Barrientos JR, Cabrera Rangel JF (2020). Narnaviruses: novel players in fungal–bacterial symbioses. ISME J.

[CR10] Eusebio Cope A, Suzuki N (2015). Mycoreovirus genome rearrangements associated with RNA silencing deficiency. Nucleic Acids Res.

[CR11] Fu RT, Chen C, Wang J (2022). Transcription profiling of rice panicle in response to crude toxin extract of *Ustilaginoidea virens*. Front Microbiol.

[CR12] Fu YJ, Wang T, Zhou SY (2023). A novel narnavirus isolated from *Colletotrichum curcumae* strain 780–2T. Arch Virol.

[CR13] Ghabrial SA, Suzuki N (2009). Viruses of plant pathogenic fungi. Annu Rev Phytopathol.

[CR14] Ghabrial SA, Castón JR, Jiang DH (2015). 50-plus years of fungal viruses. Virology.

[CR15] Guo XY, Li Y, Fan J (2012). Progress in the study of false smut disease in rice. J Agric Sci Technol.

[CR16] Guo MP, Bian YB, Wang JJ (2017). Biological and molecular characteristics of a novel partitivirus infecting the edible fungus *Lentinula edodes*. Plant Dis.

[CR17] Hafrén A, Macia JL, Love AJ (2017). Selective autophagy limits cauliflower mosaic virus infection by NBR1-mediated targeting of viral capsid protein and particles. Proc Natl Acad Sci USA.

[CR18] Hafrén A, Üstün S, Hochmuth A (2018). Turnip mosaic virus counteracts selective autophagy of the viral silencing suppressor HCpro. Plant Physiol.

[CR19] He ZR, Huang XT, Fan Y (2022). Metatranscriptomic analysis reveals rich mycoviral diversity in three major fungal pathogens of rice. Int J Mol Sci.

[CR20] Herrero N, Dueñas E, Quesada Moraga E, Zabalgogeazcoa I (2012). Prevalence and diversity of viruses in the entomopathogenic fungus beauveria bassiana. Appl Environ Microbiol.

[CR21] Hillman BI, Cai GH, Ghabrial SA (2013). The family *Narnaviridae*: simplest of RNA viruses. Advances in virus research.

[CR22] Hillman BI, Annisa A, Suzuki N, Kielian M, Mettenleiter TC, Roossinck MJ (2018). Viruses of plant-interacting fungi. Advances in virus research.

[CR23] Hollings M (1962). Viruses associated with a die-back disease of cultivated mushroom. Nature.

[CR24] Hu Z, Zheng L, Huang JB (2020). Ustiloxin A is produced early in experimental *Ustilaginoidea virens* infection and affects transcription in rice. Curr Microbiol.

[CR25] Huang XQ, Wang JK, Chen SP (2023). Rhabdovirus encoded glycoprotein induces and harnesses host antiviral autophagy for maintaining its compatible infection. Autophagy.

[CR26] Jiang DH, Fu YP, Guoqing L, Ghabrial SA, Ghabrial SA (2013). Viruses of the plant pathogenic fungus *Sclerotinia sclerotiorum*. Advances in virus research.

[CR27] Jiang YH, Luo CX, Jiang DH (2014). The complete genomic sequence of a second novel partitivirus infecting *Ustilaginoidea virens*. Arch Virol.

[CR28] Jiang YH, Zhang TT, Luo CX (2015). Prevalence and diversity of mycoviruses infecting the plant pathogen *Ustilaginoidea virens*. Virus Res.

[CR29] Kartali T, Nyilasi I, Szabó B (2019). Detection and molecular characterization of novel dsRNA viruses related to the *Totiviridae* family in *Umbelopsis ramanniana*. Front Cell Infect Microbiol.

[CR30] King AMQ, Adams MJ, Carstens EB, Lefkowitz EJ (2012). Family-*Totiviridae*. Virus taxonomy.

[CR31] Koiso Y, Li Y, Iwasaki S (1994). Ustiloxins, antimitotic cyclic peptides from false smut smut balls on rice panicles caused by *Ustilaginoidea virens*. J Antibiot.

[CR32] Kotta-Loizou I, Coutts RHA (2017). Mycoviruses in *Aspergilli*: a comprehensive review. Front Microbiol.

[CR33] Letunic I, Khedkar S, Bork P (2021). SMART: recent updates, new developments and status in 2020. Nucleic Acids Res.

[CR34] Li FF, Zhang CW, Li YZ (2018). Beclin1 restricts RNA virus infection in plants through suppression and degradation of the viral polymerase. Nat Commun.

[CR35] Li YJ, Wang M, Liu ZH (2019). Towards understanding the biosynthetic pathway for ustilaginoidin mycotoxins in *Ustilaginoidea virens*. Environ Microbiol.

[CR36] Li PF, Wang SC, Zhang LH (2020). A tripartite ssDNA mycovirus from a plant pathogenic fungus is infectious as cloned DNA and purified virions. Sci Adv.

[CR37] Lin Y, Zhou J, Zhou X (2020). A novel narnavirus from the plant-pathogenic fungus *Magnaporthe oryzae*. Arch Virol.

[CR38] Liu Y, Zhang LY, Esmael A (2020). Four novel Botourmiaviruses co-infecting an isolate of the rice blast fungus *Magnaporthe oryzae*. Viruses.

[CR39] Liu H, Wang H, Liao XL (2022). Mycoviral gene integration converts a plant pathogenic fungus into a biocontrol agent. Proc Natl Acad Sci USA.

[CR40] Liu L, Wang B, Duan GH (2023). Histone deacetylase UvHST2 is a global regulator of secondary metabolism in *Ustilaginoidea virens*. J Agric Food Chem.

[CR41] Lu SQ, Tian J, Sun WB (2014). Bis-naphtho-γ-pyrones from fungi and their bioactivities. Molecules.

[CR42] Lu SQ, Sun WB, Meng JJ (2015). Bioactive bis-naphtho-γ-pyrones from rice false smut pathogen *Ustilaginoidea virens*. J Agric Food Chem.

[CR43] Mardanov AV, Beletsky AV, Tanashchuk TN (2020). A novel narnavirus from a *Saccharomyces cerevisiae* flor strain. Arch Virol.

[CR44] Meng JJ, Gu G, Dang PQ (2019). Sorbicillinoids from the fungus *Ustilaginoidea virens* and their phytotoxic, cytotoxic, and antimicrobial activities. Front Chem.

[CR45] Mouna L, Hernandez E, Bonte D (2016). Analysis of the role of autophagy inhibition by two complementary human cytomegalovirus BECN1/Beclin 1-binding proteins. Autophagy.

[CR46] Mu F, Li B, Cheng SF (2021). Nine viruses from eight lineages exhibiting new evolutionary modes that co-infect a hypovirulent phytopathogenic fungus. PLoS Pathog.

[CR47] O’Reilly EK, Kao CC (1998). Analysis of RNA-dependent RNA polymerase structure and function as guided by known polymerase structures and computer predictions of secondary structure. Virology.

[CR48] Osaki H, Sasaki A, Nomiyama K, Tomioka K (2016). Multiple virus infection in a single strain of *Fusarium poae* shown by deep sequencing. Virus Genes.

[CR49] Segers GC, Van Wezel R, Zhang XM (2006). Hypovirus papain-like protease p29 suppresses RNA silencing in the natural fungal host and in a heterologous plant system. Eukaryot Cell.

[CR50] Segers GC, Zhang XM, Deng FY (2007). Evidence that RNA silencing functions as an antiviral defense mechanism in fungi. Proc Natl Acad Sci USA.

[CR51] Shi NJ, Xie T, Yang GG (2023). Molecular characterization of two novel totiviruses coinfecting the basal fungus *Conidiobolus adiaeretus*. Arch Virol.

[CR52] Sun Q, Choi GH, Nuss DL (2009). A single Argonaute gene is required for induction of RNA silencing antiviral defense and promotes viral RNA recombination. Proc Natl Acad Sci USA.

[CR53] Sun WX, Fan J, Fang AF (2020). *Ustilaginoidea virens*: insights into an emerging rice pathogen. Annu Rev Phytopathol.

[CR54] Thapa V, Roossinck MJ (2019). Determinants of coinfection in the mycoviruses. Front Cell Infect Microbiol.

[CR55] Vázquez AL, Alonso JMM, Parra F (2000). Mutation analysis of the GDD sequence motif of a calicivirus RNA-dependent RNA polymerase. J Virol.

[CR56] Walker PJ, Firth C, Widen SG (2015). Evolution of genome size and complexity in the *Rhabdoviridae*. PLoS Pathog.

[CR57] Wang XH, Wang J, Lai DW (2017). Ustiloxin G, a new cyclopeptide mycotoxin from rice false smut balls. Toxins.

[CR58] Wang HW, Sun SL, Ge WY (2020). Horizontal gene transfer of *Fhb7* from fungus underlies *Fusarium* head blight resistance in wheat. Science.

[CR59] Wang Q, Lu L, Zeng M (2022). Rice black-streaked dwarf virus P10 promotes phosphorylation of GAPDH (glyceraldehyde-3-phosphate dehydrogenase) to induce autophagy in *Laodelphax striatellus*. Autophagy.

[CR60] Wen H, Shi HB, Jiang N (2023). Antifungal mechanisms of silver nanoparticles on mycotoxin producing rice false smut fungus. iScience.

[CR61] Xie JT, Jiang DH (2014). New insights into mycoviruses and exploration for the biological control of crop fungal diseases. Annu Rev Phytopathol.

[CR62] Xie JM, Chen YR, Cai GJ (2023). Tree visualization by one table (tvBOT): a web application for visualizing, modifying and annotating phylogenetic trees. Nucleic Acids Res.

[CR63] Yang M, Zhang YL, Xie XL (2018). *Barley stripe mosaic virus* γb protein subverts autophagy to promote viral infection by disrupting the ATG7-ATG8 interaction. Plant Cell.

[CR64] Yang DW, He NQ, Huang FH (2023). The genetic mechanism of the immune response to the rice false smut (RFS) fungus *Ustilaginoidea virens*. Plants.

[CR65] Yu X, Li B, Fu YP (2010). A geminivirus-related DNA mycovirus that confers hypovirulence to a plant pathogenic fungus. Proc Natl Acad Sci USA.

[CR66] Zhang XM, Segers GC, Sun QH (2008). Characterization of hypovirus-derived small RNAs generated in the chestnut blight fungus by an inducible DCL-2-dependent pathway. J Virol.

[CR67] Zhang TT, Jiang YH, Huang JB, Dong WB (2013). Complete genome sequence of a putative novel victorivirus from *Ustilaginoidea virens*. Arch Virol.

[CR68] Zhang TT, Jiang YH, Huang JB, Dong WB (2013). Genomic organization of a novel partitivirus from the phytopathogenic fungus *Ustilaginoidea virens*. Arch Virol.

[CR69] Zhang TT, Jiang YH, Dong WB (2014). A novel monopartite dsRNA virus isolated from the phytopathogenic fungus *Ustilaginoidea virens* and ancestrally related to a mitochondria-associated dsRNA in the green alga *Bryopsis*. Virology.

[CR70] Zhang Y, Zhang K, Fang AF (2014). Specific adaptation of *Ustilaginoidea virens* in occupying host florets revealed by comparative and functional genomics. Nat Commun.

[CR71] Zhang R, Hisano S, Tani A (2016). A capsidless ssRNA virus hosted by an unrelated dsRNA virus. Nat Microbiol.

[CR72] Zhang TT, Zeng XX, Zeng Z (2018). A novel monopartite dsRNA virus isolated from the phytopathogenic fungus *Ustilaginoidea virens* strain GZ-2. Arch Virol.

[CR73] Zhang HX, Xie JT, Fu YP (2020). A 2-kb mycovirus converts a pathogenic fungus into a beneficial endophyte for *Brassica* protection and yield enhancement. Mol Plant.

[CR74] Zhang XP, Xu D, Hou XW (2022). *UvSorA* and *UvSorB* involved in sorbicillinoid biosynthesis contribute to fungal development, stress response and phytotoxicity in *Ustilaginoidea virens*. Int J Mol Sci.

[CR75] Zheng DW, Wang Y, Han Y (2016). *UvHOG1* is important for hyphal growth and stress responses in the rice false smut fungus *Ustilaginoidea virens*. Sci Rep.

[CR76] Zhong J, Lei XH, Zhu JZ (2014). Detection and sequence analysis of two novel co-infecting double-strand RNA mycoviruses in *Ustilaginoidea virens*. Arch Virol.

[CR77] Zhong J, Zhou Q, Lei XH (2014). The nucleotide sequence and genome organization of two victoriviruses from the rice false smut fungus *Ustilaginoidea virens*. Virus Genes.

[CR78] Zhong J, Zhu JZ, Lei XH (2014). Complete genome sequence and organization of a novel virus from the rice false smut fungus *Ustilaginoidea virens*. Virus Genes.

[CR79] Zhong J, Cheng CY, Gao BD (2017). Mycoviruses in the plant pathogen *Ustilaginoidea virens* are not correlated with the genetic backgrounds of its hosts. Int J Mol Sci.

[CR80] Zhou SY, Chen DP, Fu YJ (2023). Characterization of a novel mycotombus-like virus from the plant-pathogenic fungus *Phoma matteucciicola*. Arch Virol.

[CR81] Zhu HJ, Chen D, Zhong J (2015). A novel mycovirus identified from the rice false smut fungus *Ustilaginoidea virens*. Virus Genes.

