# Supplementary material for: Co-infection of Four Novel Mycoviruses from Three Lineages Confers Hypovirulence on Phytopathogenic Fungus Ustilaginoidea virens
Source: Rice (N Y). 2024 Jul 16;17:44. doi: 10.1186/s12284-024-00721-z (PMC11252108; doi:10.1186/s12284-024-00721-z)
Supplement: Supplementary file 1 — Additional file 1. [file 12284_2024_721_MOESM1_ESM.docx]

| **Primer Name** | **Sequence (5'---3')** | **Direction** | **Used for** |
| --- | --- | --- | --- |
| **UvRV16-F** | TGTTAGGGCGAGGGGTAGGG | Forward | UvRV16 RT-PCR |
| **UvRV16-R** | GCACAATGTGGAGTGATTGCCA | Reverse |  |
| **UvRV16 5'RACE inner_F** | CGTGCTTAGAAAAGCTGCCG | Forward | UvRV16 5' RACE |
| **UvRV16 5'RACE outer_F** | TCGAAGAGCGAGCTCATTACCG | Forward |  |
| **UvRV16 3'RACE inner_R** | TGACTACGCTACACTGTTGACGC | Reverse | UvRV16 5' RACE |
| **UvRV16 3'RACE outer_R** | CAAGCAGAGCGTGGGTACTG | Reverse |  |
| **UvNV13-F** | CTGCCTGGGGGTTACCAGG | Forward | UvBV13 RT-PCR |
| **UvNV13-R** | GGATCAAAGCCGAGCTGCG | Reverse |  |
| **UvNV13 5'RACE inner_F** | TCAAAGGCAGCGACGCGCCTGGCT | Forward | UvBV13 5' RACE |
| **UvNV13 5'RACE outer_F** | GCTCAGAGTCACGCTGCCGATCAAAGG | Forward |  |
| **UvNV13 3'RACE inner_R** | TTGCTCGGGGCCTATGGTCTCTCG | Reverse | UvBV13 5' RACE |
| **UvNV13 3'RACE outer_R** | TACCGTTATGGACCCCGAGTCTTGCTCG | Reverse |  |
| **UvBV8-F** | CGGTTGGCATGAGGTGACC | Forward | UvBV13 RT-PCR |
| **UvBV8-R** | GGACACGCTCCACAAGCTAGT | Reverse |  |
| **UvBV8 5'RACE inner_F** | CGTTGACAAGGAAGCTCGCT | Forward | UvBV13 5' RACE |
| **UvBV8 5'RACE outer_F** | TACCCGAGGGGTAGGATGACG | Forward |  |
| **UvBV8 3'RACE inner_R** | CAGATCGCAAGCGAGTGAGG | Reverse | UvBV13 5' RACE |
| **UvBV8 3'RACE outer_R** | ACAAAGGAAATGAGGGCGATCC | Reverse |  |
| **UvBV9-F** | TCTTGCCCACCGAGTTCGTT | Forward | UvBV13 RT-PCR |
| **UvBV9-R** | TCACCAGAGAGAGGCTCCCTC | Reverse |  |
| **UvBV9 5'RACE inner_F** | AGGATGAAATCCTTACGAGCCCCGAGCC | Forward | UvBV13 5' RACE |
| **UvBV9 5'RACE outer_F** | TCCTCCTATAAACCGACGGGTCCTTCCC | Forward |  |
| **UvBV9 3'RACE inner_R** | GTCGTCAACGTCAAGCCGAG | Reverse | UvBV13 5' RACE |
| **UvBV9 3'RACE outer_R** | CTCTCTCTGGTGAGAGGGCAC | Reverse |  |

**S1 Table. The primer information for the detection and RACE of four mycoviruses.**
