# Supplementary material for: Co-infection of Four Novel Mycoviruses from Three Lineages Confers Hypovirulence on Phytopathogenic Fungus Ustilaginoidea virens
Source: Rice (N Y). 2024 Jul 16;17:44. doi: 10.1186/s12284-024-00721-z (PMC11252108; doi:10.1186/s12284-024-00721-z)
Supplement: Supplementary file 2 — Additional file 2. [file 12284_2024_721_MOESM2_ESM.docx]

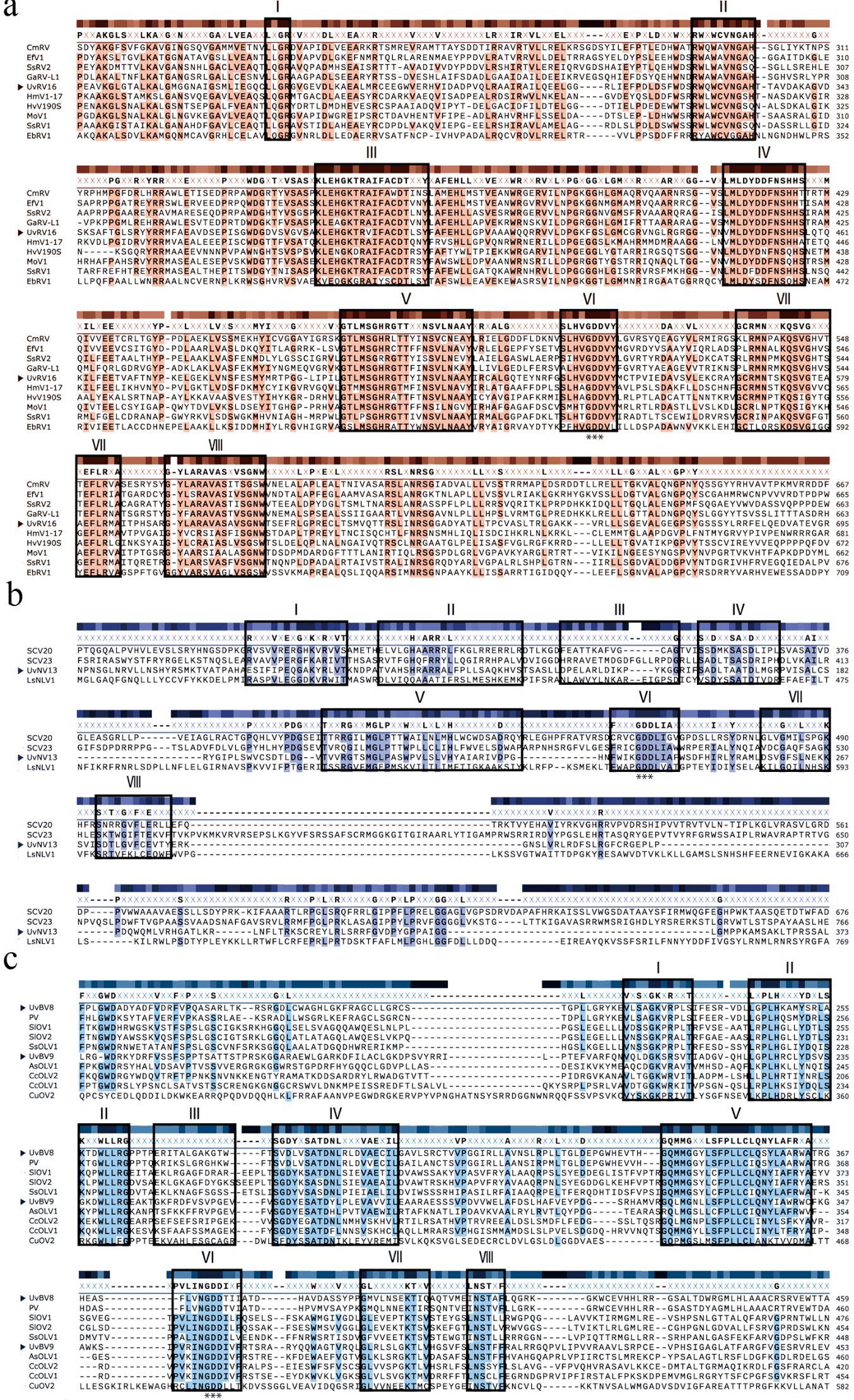


**Fig. S1 Sequence alignments of four potentially novel mycoviruses.**

(a) Alignment based on the RdRP amino acid sequences of UvRV16 and other viruses of the same genus was performed to analyses conserved structural domains in the protein sequences. (b) Alignment based on the RdRP amino acid sequences of UvNV13 and other viruses of the same genus was performed to analyses conserved structural domains in the protein sequences. (c) Alignment based on the RdRP amino acid sequences of UvBV8, UvBV9 and other viruses of the same genus was performed to analyses conserved structural domains in the protein sequences. Conserved motifs in the RdRP of comparative viruses are indicated by Roman numerals I to VIII. Identical amino acid residues are highlighted in darker colors, while conserved and semi-conserved residues are shown in lighter colors. The "_***_" symbol in structural domain VI shows the highly conserved “GDD” motifs in the virus.


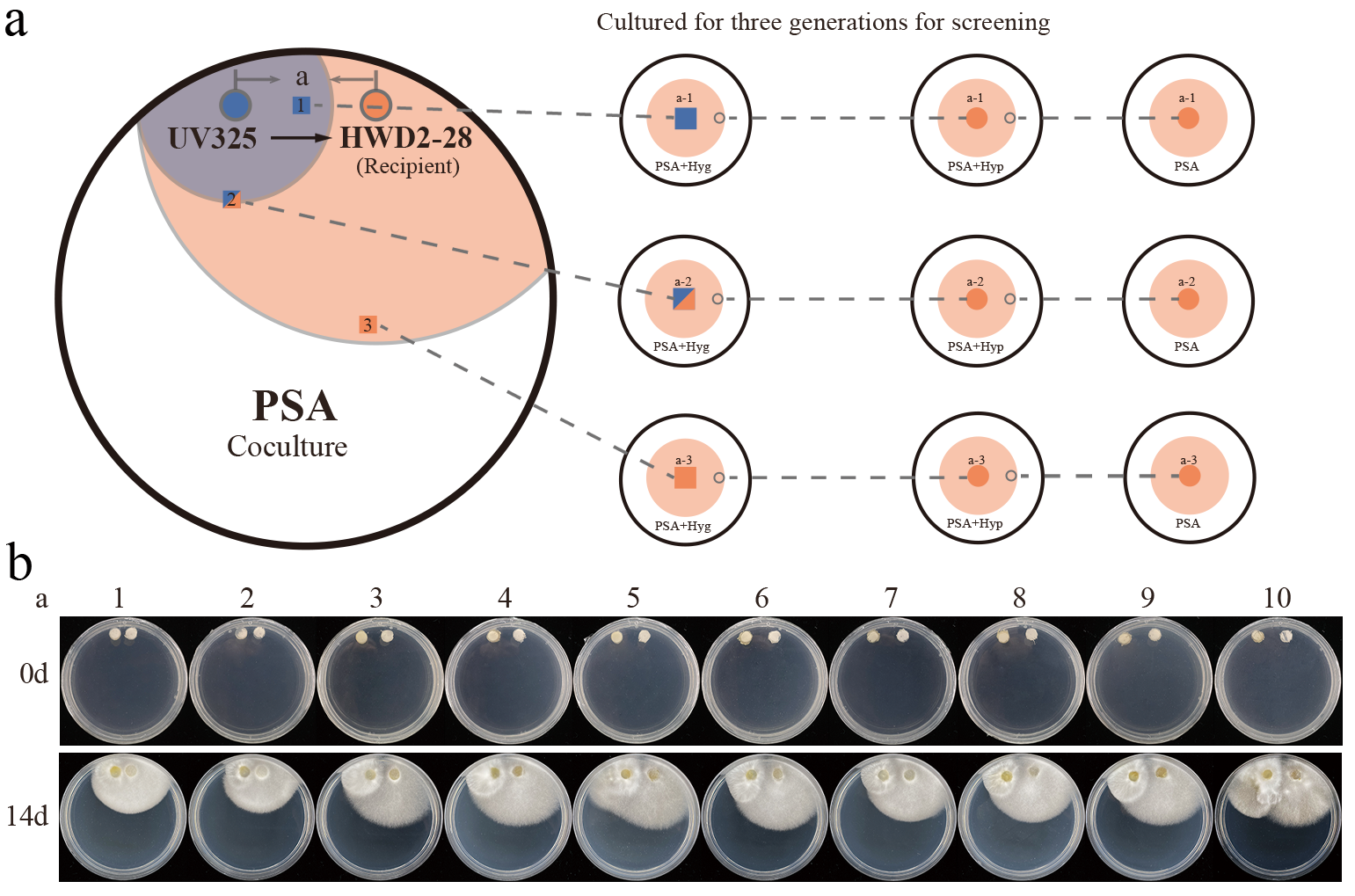


**Fig. S2 Horizontal transmission of mycoviruses.**

(a) Schematic representation of the horizontal transmission system of mycoviruses. Virus-infected strain Uv325 (donor strain) was co-cultured with recipient strain HWD2-28 on PSA medium plates with mycelial plugs placed at increasing spacing "a". After waiting for mycelial homozygosity, mycelia were picked from the donor strain, the recipient strain and the mycelial fusion region, respectively, and transferred to PSA medium supplemented with hygromycin B and cultured for three generations at 28°C in the dark in order to screen for homozygous strains of HWD2-28. (b) Colony morphology of Uv325 co-cultured with HWD2-28 in PSA medium at 28°C in the dark on days 0 and 14.


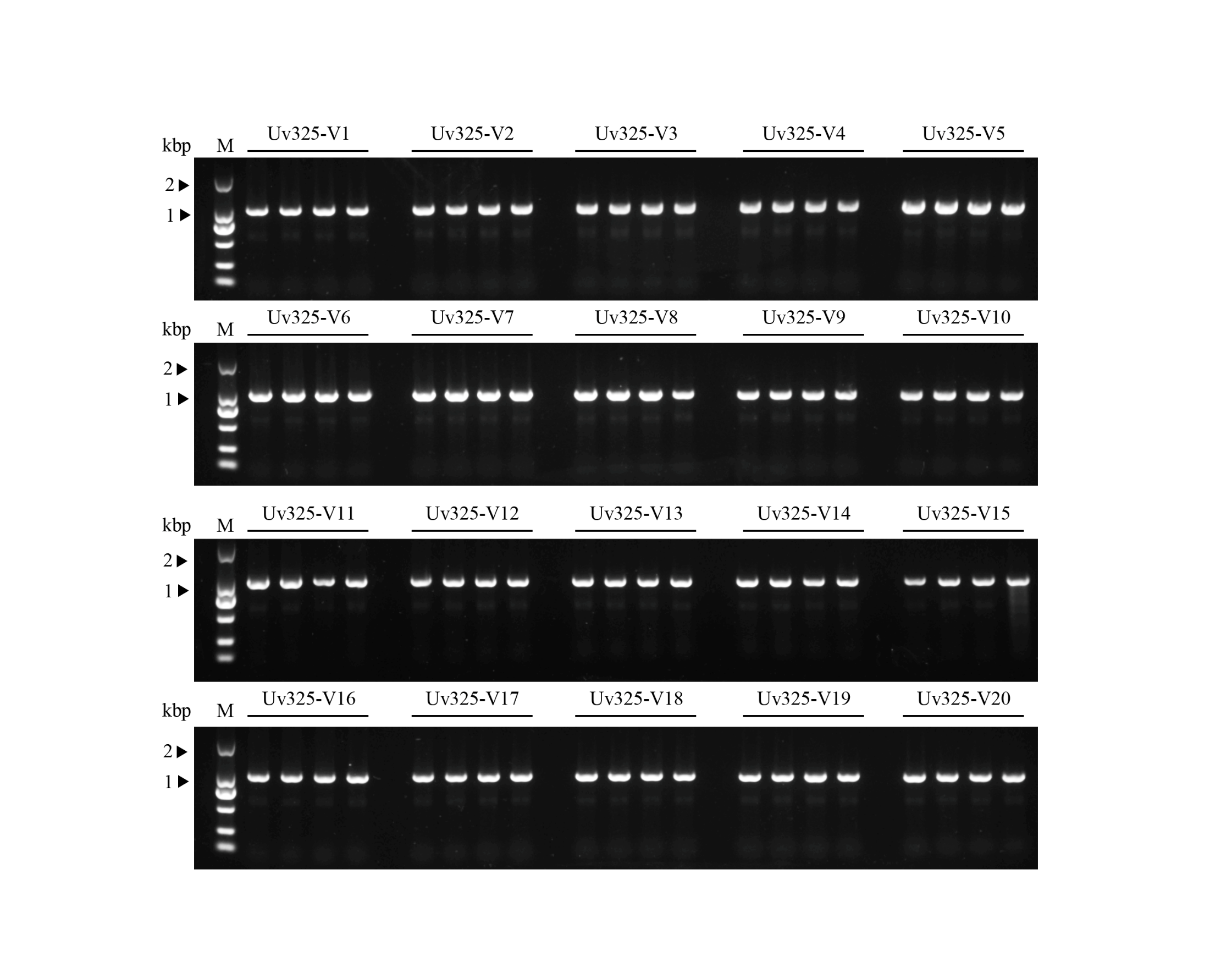


**Fig. S3 Vertical transmission of mycoviruses.** Individual conidia were isolated from the mycelium of *U. virens* strain Uv325 and cultured on PSA, and a total of 20 subisolates were randomly selected, and RT-PCR was analyzed for the presence of the four mycoviruses. Lane M: DL2000 DNA molecular weight marker.
